# Lead-Free Perovskite Thin Films with Tailored Pockels-Kerr Effects for Photonics

**DOI:** 10.1021/acsami.3c06499

**Published:** 2023-07-27

**Authors:** Valentin Ion, Valentin Teodorescu, Ruxandra Birjega, Maria Dinescu, Christoph Mitterbauer, Ioannis Alexandrou, Ioan Ghitiu, Floriana Craciun, Nicu D. Scarisoreanu

**Affiliations:** † National Institute for Laser, Plasma and Radiation Physics, 409 Atomistilor, Magurele 077125, Romania; ‡ National Institute of Materials Physics, 105 bis Atomistilor, Magurele 077125, Romania; § Thermo Fisher Scientific, Materials & Structural Analysis, De Schakel 2, Eindhoven 5651 GE, the Netherlands; ∥ Faculty of Physics, University of Bucharest, Magurele 077125, Romania; ⊥ CNR-ISM, Istituto di Struttura della Materia, Area della Ricerca di Roma-Tor Vergata, Via del Fosso del Cavaliere 100, Rome I-00133, Italy

**Keywords:** thin films, lead-free perovskites, nanopolar domains, optical nonlinearities, electro-optic effects

## Abstract

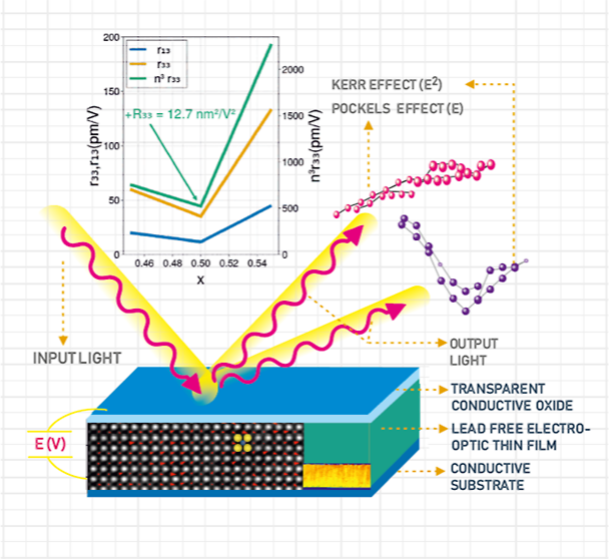

Pockels and Kerr effects are linear and nonlinear electro-optical effects, respectively, used in many applications. The modulation of the refractive index is employed in different photonic circuits. However, the greatest challenge is in photonic elements for quantum computing at room temperature. For this aim, materials with strong Pockels/Kerr effects and χ^(2)^/χ^(3)^ nonlinear susceptibilities are necessary. Here, we demonstrate composition-modulated strong electro-optical response in epitaxial films of (Ba,Ca)(Ti,Zr)O_3_ perovskite titanate. These films are grown by pulsed laser deposition on SrTiO_3_. Depending on the ratios of Ca/Ba and Ti/Zr, films show high Pockels or Kerr optical nonlinearities. We relate the variable electro-optic response to the occurrence of nanopolar domains with different symmetries in a selected composition range. These findings open the route to easily implement nonlinear optical elements in integrated photonic circuits.

## Introduction

A noncentrosymmetric medium can exhibit a variation of its refraction index upon the application of an electric field, the response being known as the Pockels effect if linear and the Kerr effect if nonlinear.^1^ These electro-optic effects have been intensively studied in the last decade for use in high-speed electro-optical modulators and switches for integrated photonics applications,^2^ photonic integrated circuits for room-temperature quantum computing,^3−5^ and electro-optic devices for coherent conversion between microwave and optical photons.^4^ The electronic parts of Pockels/Kerr effects (χ^(2)^/χ^(3)^ nonlinearities) are involved in second and third harmonic generations^6,7^ which are at the base of quantum optical computing architectures.^8−10^ The standard material for use in optical modulation is LiNbO_3_.^11^ However, LiNbO_3_ is not integrable on Si,^12^ therefore perovskite titanates became the focus of recent theoretical^13^ and experimental^14^ studies. Different results have been reported concerning BaTiO_3_ thin-film-based modulators and other electro-optic devices.^15−21^

It becomes therefore very important to find materials with enhanced electro-optic coefficients and, generally, with strong optical nonlinearities, high dielectric susceptibility, low loss, and high refractive index in order to optimize the integrated electro-optic devices. It has been theoretically shown that the mechanism that drives the high electro-optic response of BTO involves a large Raman susceptibility (strong electron–phonon coupling) and soft phonons,^13^ which explains why perovskite titanates, which fulfill both requirements, are at the center of recent research.^14^

Among the perovskite titanates, (Ba,Ca)(Ti,Zr)O_3_ (BCTZ) has been singled out as the most promising ferroelectric among lead-free ferroelectric perovskites for piezoelectric applications.^21^ (Ba,Ca)(Ti,Zr)O_3_ is a ferroelectric perovskite with strong ferroelectric and piezoelectric properties.^21^ Unlike other ferroelectrics traditionally used in devices, like Pb(Zr,Ti)O_3_, (Pb,La)(Zr,Ti)O_3_, Pb(Mg_1/3_ Nb_2/3_)O_3_–PbTiO_3_, etc., it contains only safe and bio-compatible elements,^22^ and none of its elements are rare. To understand better its structural and functional properties, this solid solution has been often rationalized in terms of two end members: Ba(Ti_0.8_Zr_0.2_)O_3_ (BTZ) and (Ba_0.7_Ca_0.3_)TiO_3_ (BCT) and it is usually written as (1 – *x*)BTZ – *x*BCT (BCTZ 100×).^23^ This solid solution has a morphotropic phase boundary (MPB) between a rhombohedral *R*3*m* and a tetragonal *P*4*mm* phase, with an intermediate orthorhombic *Amm*2 phase, at *x* ≅ 0.5.^23−25^ Microscopically, from a first-principles study it has been assessed that both competition between different polar phases driven by the B-type (Ti) and A-type (Ca) ferroelectricity, as well as partial destabilization of ferroelectric phase by Zr-substitution (and occurrence of polar nanoregions in Ti-rich regions) are present, possibly enhancing the piezoelectric response.^26^ At MPB, the BCTZ functional properties (dielectric and elastic susceptibilities, piezoelectric coefficients, ferroelectric properties, etc.) are maximized, due to the coexistence of different phases, even at the nanoscale.^26−28^ However, its electro-optical properties have never been explored. To understand the electro-optic effect in BCTZ with different compositions, one must explore the relationship between the microscopic properties such as lattice anharmonicity, strength of electron–phonon coupling, and the Pockels/Kerr effects. It has been shown already that BCTZ can be grown on different substrates by pulsed laser deposition (PLD).^29−31^ PLD can be employed for the growth of films on large surfaces.

We show in this work that BCTZ films with various compositions can be epitaxially grown on SrTiO_3_. We show that, depending on the ratio of Ba/Ca and Ti/Zr, films show high Pockels (χ^(2)^) or Kerr (χ^(3)^) optical nonlinearities. We show that the linear and nonlinear electro-optical properties can be tailored by changing the ratio of Ba/Ca and Ti/Zr in the composition. Their linear properties are superior to LiNbO_3_ which is the material used for optical applications. Moreover, LiNbO_3_ is difficult to be grown as thin films (no epitaxial deposition has been reported). In this work, we report BCTZ thin films with variable BCT(*x*) content (which ultimately translates into variable Ba/Ca and Ti/Zr ratios) which show linear EO properties for rhombohedral (*x* = 0.45) and tetragonal (*x* = 0.55) compositions and nonlinear EO properties for MPB composition (*x* = 0.50), where a mix of phases coexist at the nanoscale, rending it similar to a relaxor ferroelectric with nanopolar regions. The quadratic EO effect is attributed to the coexistence of nanopolar regions with different symmetries. These findings open the way to the efficient integration of different electro-optical devices (modulators, tuning elements, bistable switches, etc.), including χ^(2)^- and χ^(3)^-based elements for room-temperature photonic quantum processing.^5,9,10^

## Results and Discussion

### Structural Characterization

**Figure 1:**
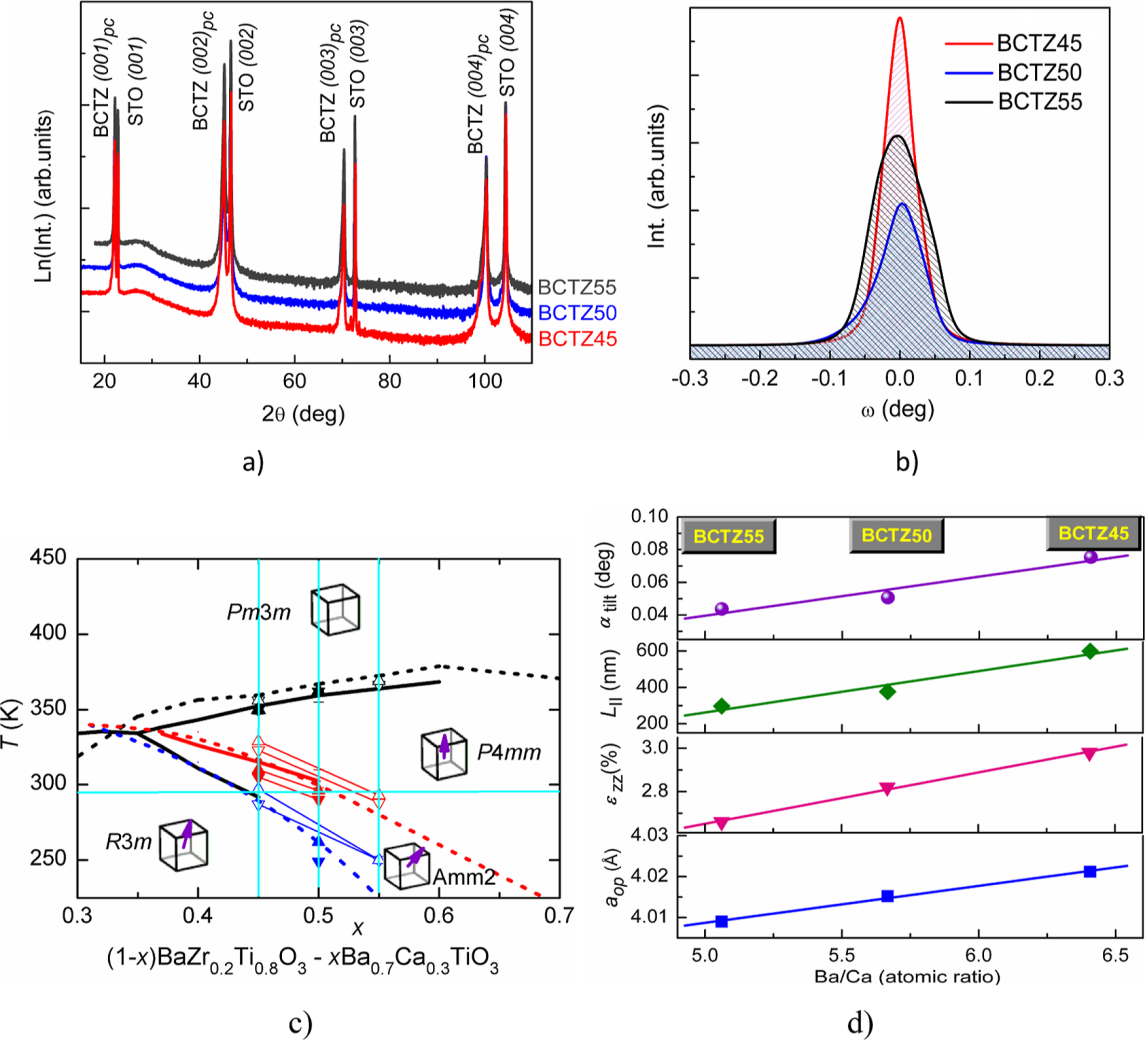
XRD characterization of BCTZ films. (a) XRD patterns of the BCTZ films with different compositions deposited on the STO(001) substrate, plotted in log scale; (b) rocking curves (ω-scans) of the symmetric (002)_pc_ reflection; and (c) phase diagram of BCTZ solid solution, modified from ref (25). The intersections of the light-blue lines mark the positions of BCTZ film compositions. (d) Linear dependence of some structural parameters with the Ba/Ca atomic ratio of BCTZ films.

The X-ray diffraction (XRD) patterns of the BCTZ films with different chemical compositions are shown in Figure 1a. The θ*–*2θ scans show only (00*l*) reflections with no additional phases or orientations, consistent with an epitaxial growth onto the STO(001) substrate. The epitaxy of BCTZ films was confirmed by the BCTZ(101) and STO(101) Φ-scans (Supporting Information, Figure S2), perfectly matching a fourfold symmetry with 90° spacing for all the three films. The patterns were indexed as a pseudocubic lattice.

The out-of-plane (*a*_op_) and in-plane (*a*_ip_) lattice parameters were calculated as explained in Supporting Information, Note 1, from symmetric and asymmetric XRD scans, respectively. The lattice parameters *a*_op_ and *a*_ip_, the axial ratio (or tetragonal distortion) *a*_op_/*a*_ip_, and the out-of-plane and in-plane strains due to the misfit with respect to the substrate are listed in Table 1.

**Table 1:** Structural Parameters of BCTZ Films

films	thickness (nm) SE/TEM	*a*_op_^a^(Å)	*a*_ip_^b^(Å)	*a*_op_/*a*_ip_	ε_zz_ (%)	ε_xx_ (%)	ω(002) (deg)	L_II_^c^(nm)	α_tilt_^c^(deg)
BCTZ45	703/700	4.0212 ± 0.0026	4.0262 ± 0.0037	0.999	2.98	3.1	0.0565	599 ± 57	0.0754 ± 0.0052
BCTZ50	705/700	4.0152 ± 0.0012	3.9776 ± 0.0065	1.009	2.82	1.86	0.0779	376 ± 84	0.0507 ± 0.0049
BCTZ55	600/580	4.0090 ± 0.0041	4.0130 ± 0.0009	0.999	2.66	2.76	0.0873	297 ± 92	0.0437 ± 0.0103

a Errors represent the standard error of the mean value calculated from the positions of the four (00*l*) reflections for each sample.

b Errors are extracted from the standard deviation of intercept of linear regression of *d*_*hkl*_ values of asymmetric (*hkl*) reflections against (sin ψ)^2^ plots, ψ being the tilted angle corresponding to each asymmetric (*hkl*) plane used.

c Errors are extracted from the standard deviation of intercept (for *L*_II_) and the standard deviation of the slope (for α_tilt_) from the linear regression of W–H plots derived from the ω-scans around the same (00*l*) reflections for each sample.

An examination of the data reveals close values for *a*_op_ and *a*_ip_ for all three BCTZ films. Consequently, the tetragonal distortions are almost equal to 1. It is a result of BCTZ films’ full relaxation with increasing thickness. Indeed, at such large thicknesses (600–700 nm, Table 1) the strain from the substrate is almost completely relaxed and the lattice parameters can return approximately to the bulk values. However, it must be mentioned that the strain relaxation has a non-trivial influence on the film structure, as it was evidenced in refs (30)(32)(33), where nanoscale domain morphology has been reported. Although we do not have an ultimate answer, we suspect that the vicinity of the films’ compositions to the MPB and the presence of nanodomains are important factors that favor the epitaxial growth even in such a high thickness range for the epitaxial growth. In fact, in ref (34) epitaxial films with thicknesses of up to 800 nm are obtained from compositions of BiFeO_3_–BaTiO_3_–SrTiO_3_ solid-solution deposited by PLD on STON substrates. A polymorphic nanodomain structure has been obtained for MPB compositions, and the enhanced functional properties of these films have been explained by the elimination of macroscopic domain walls and the high dynamics of nanodomains. Although it is not explicitly stated, it is possible that the presence of nanodomains also favors the preservation of epitaxial structure up to high thickness. A dense pattern of nanometric domains has also been evidenced in epitaxial PZT films with MPB PbZr_0.52_Ti_0.48_O_3_ composition.^35^

The “mosaicity” of the films was analyzed by performing ω-scans (rocking-curves) around the (00*l*)_pc_ reflections. The excellent crystallinity of the films is revealed by the small values of the (00*l*) peaks broadness of the rocking curves, like e.g., for the (002)_pc_ reflection (Figure 1b and Table 1).

The lateral coherence length (*L*_||_) and the mean mosaic tilt angle (α_tilt_) have been obtained using a Williamson–Hall (W–H) approach based on comparing the peak widths of reflections of successive orders. The lateral coherence length, *L*_||_, represents the dimension of the mosaic blocks parallel to the substrate plane, while the mean mosaic tilt angle, α_tilt_, the misorientation out of the film plane. The method is described in detail in previous studies, refs (30) and (33). It implies a “mosaic” model of the film by assuming that the epitaxial growth of the films possessing large lattice mismatch consists of oriented mosaic blocks that coherently scatter X-rays. A W–H plot of (β_ω_ sin θ)/λ versus sin θ/λ yields *L*_||_ from the intercept and α_tilt_ from the slope. Here, β_ω_ represents the FWHM of each (00*l*) rocking curve, θ is the Bragg angle, and λ the X-ray wavelength. The thickness of the films was determined both by spectrometric ellipsometry (SE) and high-resolution transmission electron microscopy (HR-TEM).

The calculated parameters, presented in Table 1, reveal large lateral coherence lengths and very small mean mosaic angles. The BCTZ45, BCTZ50, and BCTZ55 films exhibit similar structural characteristics due to their proximity to the MPB, their higher thickness, and lattice misfit strain. This can be better appreciated from Figure 1c, where a sketch of the BCTZ phase diagram is shown. The intersection of the three vertical lines with the horizontal line at room temperature represent the three compositions which have been investigated here. It can be observed that, at room temperature, BCTZ45 is near the R–O phase boundary, BCTZ55 is near the O–T phase boundary, while BCTZ50 is in the O region, in-between the R and T phases.

Although small, there is an observable effect of the partial substitution of Ba^2+^ by Ca^2+^ on the A sites (Figure 1d). Thus, substituting the smaller Ca^2+^ ion decreases the unit cell size, as reflected in the out-of-plane lattice parameter decreasing with the Ba/Ca ratio decreasing. Moreover, a smaller strain (ε_*zz*_) due to the misfit lattice differences between the film and the substrate along the growth direction is observed at a smaller Ba/Ca ratio. Also, a smaller coherence length due to higher induced cation disorder is observed in films with higher content of Ca (as in the BCTZ55 film).

### Mapping Nanoscale Local Structure

At room temperature, ferroelectric BCTZ exhibits a rhombohedral *R*3*m*, an orthorhombic *Amm*2, or a tetragonal *P*4*mm* phase (or a mix of them), depending on the composition.^23−25^ For the compositions investigated here (BCTZ45, BCTZ50, and BCTZ55), the lattice parameters measured on the targets are listed in Supporting Information, Table S1. In the BCTZ unit cell, the Ti^4+^ ion and the octahedron of O^2–^ ions are displaced from the center of the Ba/Ca sublattice along the directions allowed by symmetry (the diagonal of the unit cell for *R*3*m*, the face diagonal for *Amm*2, or the tetragonal axis for *P*4*mm*), resulting in a spontaneous polarization along those directions. Thus, the shift of Ti^4+^ ion, δ_Ti_, can be used to visualize the polarization direction in the ferroelectric BCTZ films. These displacements are schematically rendered in the unit cells pictured in Figure 1c.

**Figure 2:**
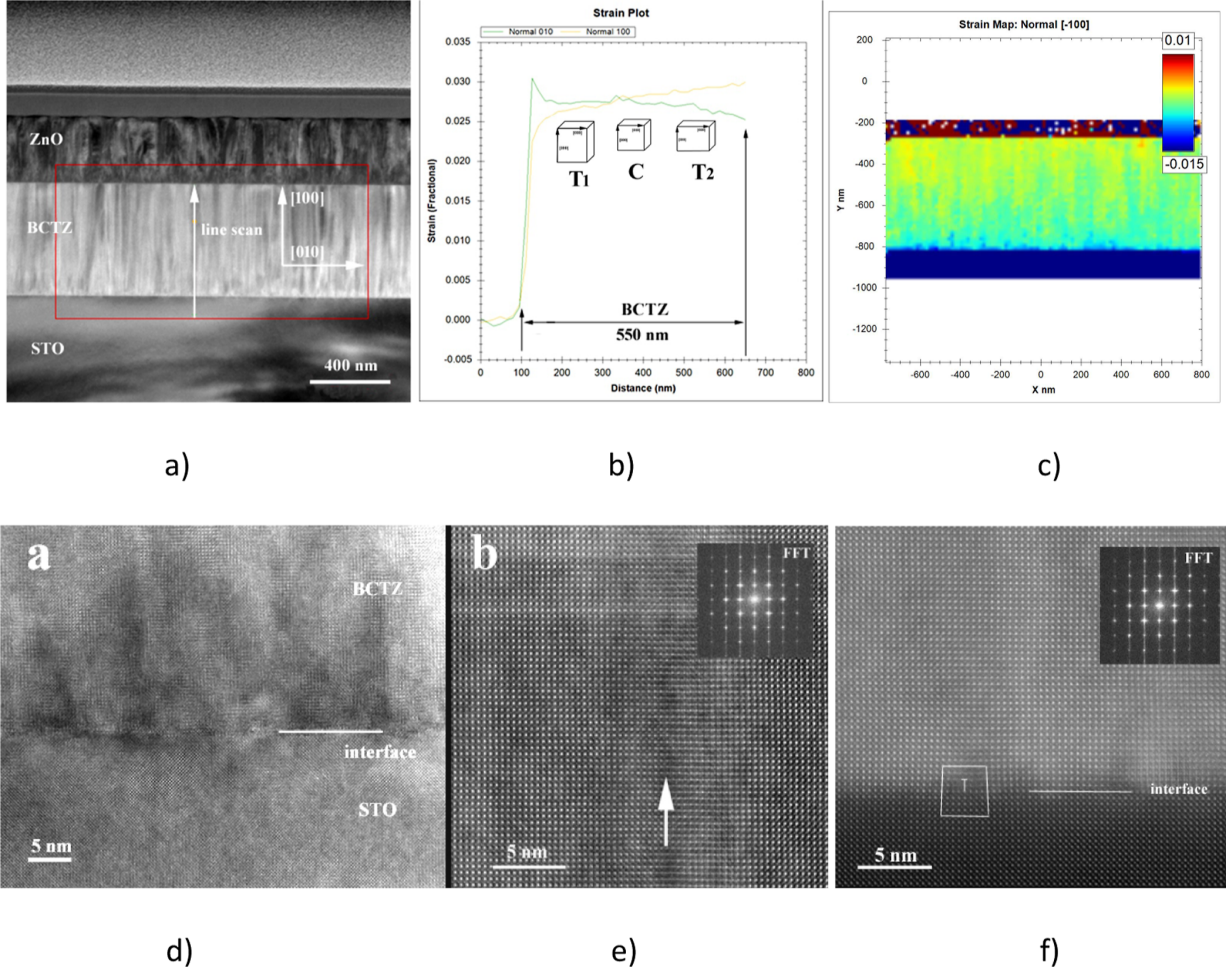
STEM characterization and strain distribution maps of the BCTZ55 film. (a) Low-resolution STEM image for the BCTZ55 film grown on STO; (b) strain profile along the vertical of the film for the in-plane (ε_*xx*_) and out-of-plane (ε_*zz*_) strain for the BCTZ55 film; (c) GPA out-of-plane strain map corresponding to the rectangle area marked in (a); (d) HR-TEM image at the interface with the substrate, marked by a line; (e) high-resolution STEM image in the middle of the film, showing the absence of defects between coherent columnar zones; and (f) high-resolution STEM image at the interface with the substrate, evidencing the presence of some dislocations, due to the misfit.

Images on the cross section of BCTZ films are acquired in the high-angle annular dark-field (HAADF) mode in aberration-corrected scanning transmission electron microscopy (STEM). A low-resolution STEM image for the BCTZ55 film grown on STO is shown in Figure 2a, together with the geometric phase analysis (GPA) image for the strain (Figure 2c) along the [100]_pc_ direction (the strain map for the [010] direction is discussed in Supporting Information, Note 2). In what follows, for simplicity, the unit cells for the different structures (R, T, and O) are treated as a pseudocubic unit cell (Supporting Information, Note 1). The upper layer in the STEM image is the Al-doped zinc oxide (AZO) conducting layer. The GPA strain maps corresponding to the rectangle area marked in the STEM image evidence a nanoscale strain variation, superposed on an inhomogeneous strain, stronger near the interface film/substrate and more relaxed near the top surface.

Strain profiles along the vertical of the film for in-plane and out-of-plane strains show that the misfit strain relaxes from the interface with the substrate toward the top surface (Figure 2b). The strain variation, which is related to the unit cell variation, shows that the unit cell dimensions and orientation change from a tetragonal cell with the tetragonal axis out-of-plane near the substrate to a nearly cubic cell in the middle and furthermore to a tetragonal cell with the in-plane axis toward the top surface, as pictured in Figure 2b.

Figure 2d shows a HR-TEM image on cross section at the interface of BCTZ55/STO, where the interface is marked by a line. Some dislocations due to misfit are visible on the HR-STEM image in the same region (Figure 2f). Figure 2e displays a HR-STEM image in the middle of the film, showing the absence of defects between different coherent columnar zones.

**Figure 3:**
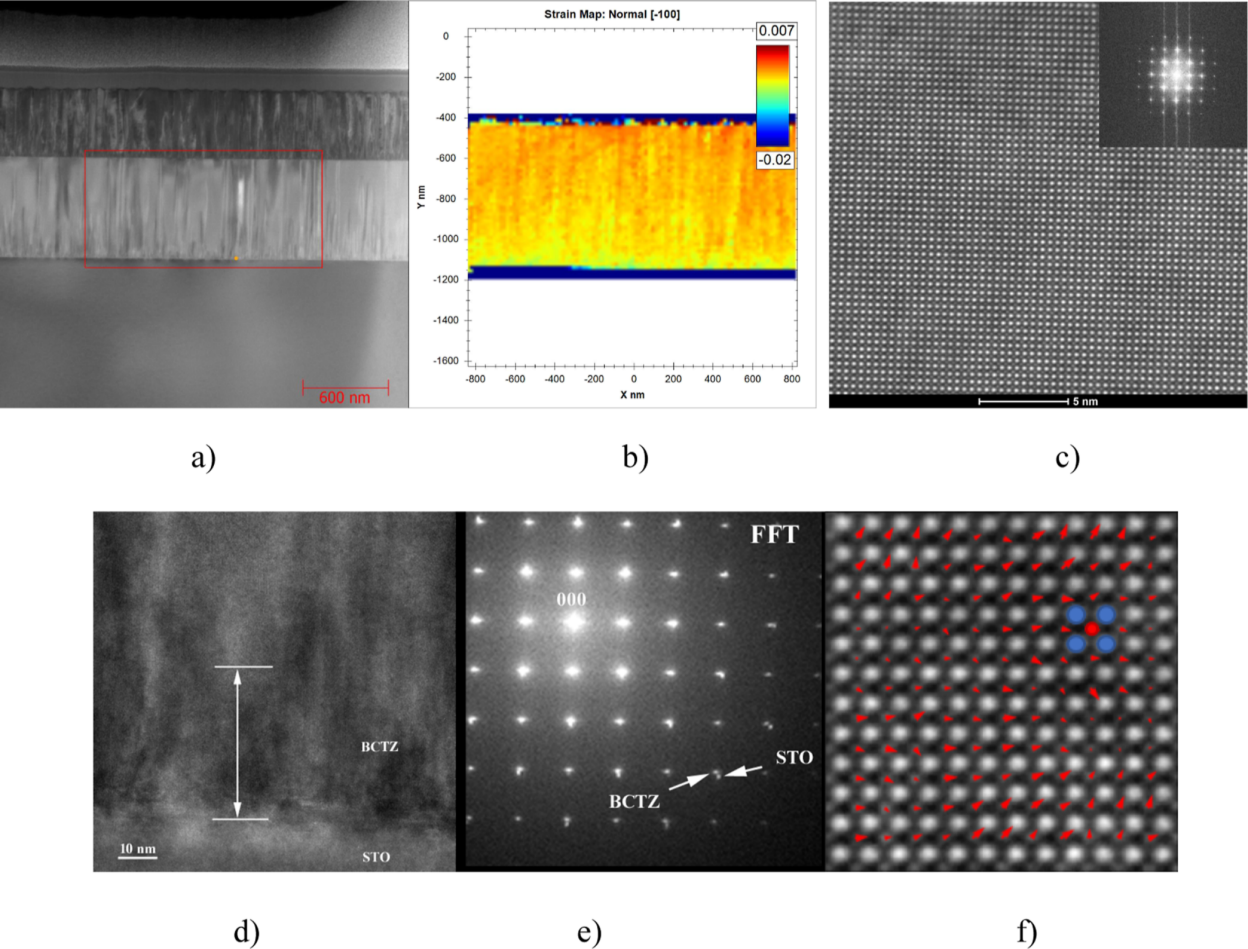
STEM characterization and strain distribution maps of the BCTZ50 film. (a) Low-resolution STEM image for the BCTZ50 film grown on STO; (b) GPA out-of-plane strain map corresponding to the rectangle area marked in (a); (c) high-resolution STEM image in the middle of the film, with FFT image in the inset; (d) HR-TEM image at the interface with the substrate; (e) FFT image evidencing the epitaxial structure; and (f) atomic-resolution STEM image of the BCTZ50 film. The red arrows show the displacement of Ti ions within the Ba/Ca cages (scaled by a factor of 5 to increase visibility). Some of the central ions shift on different directions allowed by symmetry, while others seem to not shift at all, indicating a cubic cell or a Zr ion. Ba/Ca ions are shown in blue while Ti/Zr ions in red on the projected structure.

Figure 3a shows a low-resolution STEM image for the BCTZ50 film grown on STO. The GPA out-of-plane strain map corresponding to the rectangle area marked in (a) is shown in Figure 3b. This evidences a finer nanoscale strain variation than for the BCTZ55 film, correlated with the coexistence of R, O, and T structures in this composition. A high-resolution STEM image (Figure 3c) taken in the middle of the film evidences the crystallinity of the film. The HR-TEM image taken at the BCTZ50/STO interface together with the fast Fourier transform (FFT) image confirms the epitaxial structure (Figure 3d,e).

Figure 3f shows an atomic-resolution HAADF–STEM image of the BCTZ50 film. The blue and red circles represent the positions of Ba/Ca and Ti/Zr atom columns, respectively. Ca and Zr are present in small amounts in the columns, therefore the contrast is conditioned by the majority elements. Heavy Ba^2+^ columns are much brighter than the light Ti^4+^ columns because the intensity of atom columns in STEM is approximately proportional to the square of the atomic number.

The different red arrows show the direction of displacement of Ti^4+^ ions within the Ba/Ca cages, the displacement being obtained as follows: first, the positions of the A cations were identified and fitted, based on the center of mass, being then refined using 2D Gaussian fitting and the zone axes for this sublattice were determined. Next, the positions of the B cations were inferred using the symmetry of the structure. For more robust fitting of these positions, the A cations were removed from the image using the previously mentioned Gaussian fitting, then the positions were refined using the same center of mass and 2D Gaussian fitting approach. Finally, the displacements of the B cations were calculated based on the determined positions and the symmetry-derived ones. It can be observed that clusters of Ti^4+^ ions shift along the diagonal of the cage, while others shift along the edge. There are also Ti ions which seem to not be displaced from the cell center, possibly indicating a cubic cell or a Zr-rich column, since Zr ion does not shift in the unit cell.

Thus, besides the strain map which indicates a fine nanoscale strain variation, correlated with the presence of nanodomains, the atomic resolution STEM images also evidence the presence of nanodomains in BCTZ50 films.

In Supporting Information, Note 2, the strain map of the BCTZ45 film has been discussed (Supporting Information, Figure S5). It shows a fine nanoscale strain variation, superposed on an inhomogeneous strain, similar to the strain map of the BCTZ55 film. We recall that, while in bulk ceramics MPBs are obtained by chemical substitution, in epitaxial thin films the strain engineering can stabilize new phases and ferroelectric domains. Previously it has been shown that, by increasing the BCTZ45 film thickness beyond about 100 nm, the strain relaxation occurs through an MPB-like phase mixture.^30,31^ This gives rise to a nanoscale domain structure, as evidenced by GPA. However, the richness of phases involved in the relaxed film structure is evidently higher in the case of “true” chemically induced MPB composition like BCTZ50, where the two agents (chemical strain and mechanical strain) act simultaneously, resulting into a much finer nanoscale structure, as shown also by a comparison of the GPA strain maps in Supporting Information, Figures S3–S5.

Compositional analysis and elemental mapping on BCTZ films have been obtained by Super-X energy-dispersive X-ray spectroscopy, which allows to acquire large area elemental maps with high spatial resolution and also light element sensitivity (Supporting Information, Note 3 and Figures S8–S11). The measurements demonstrate uniform distribution of the elements, with no diffusion at the interfaces.

### Dielectric Permittivity and Loss for BCTZ Films with Different Compositions

**Figure 4:**
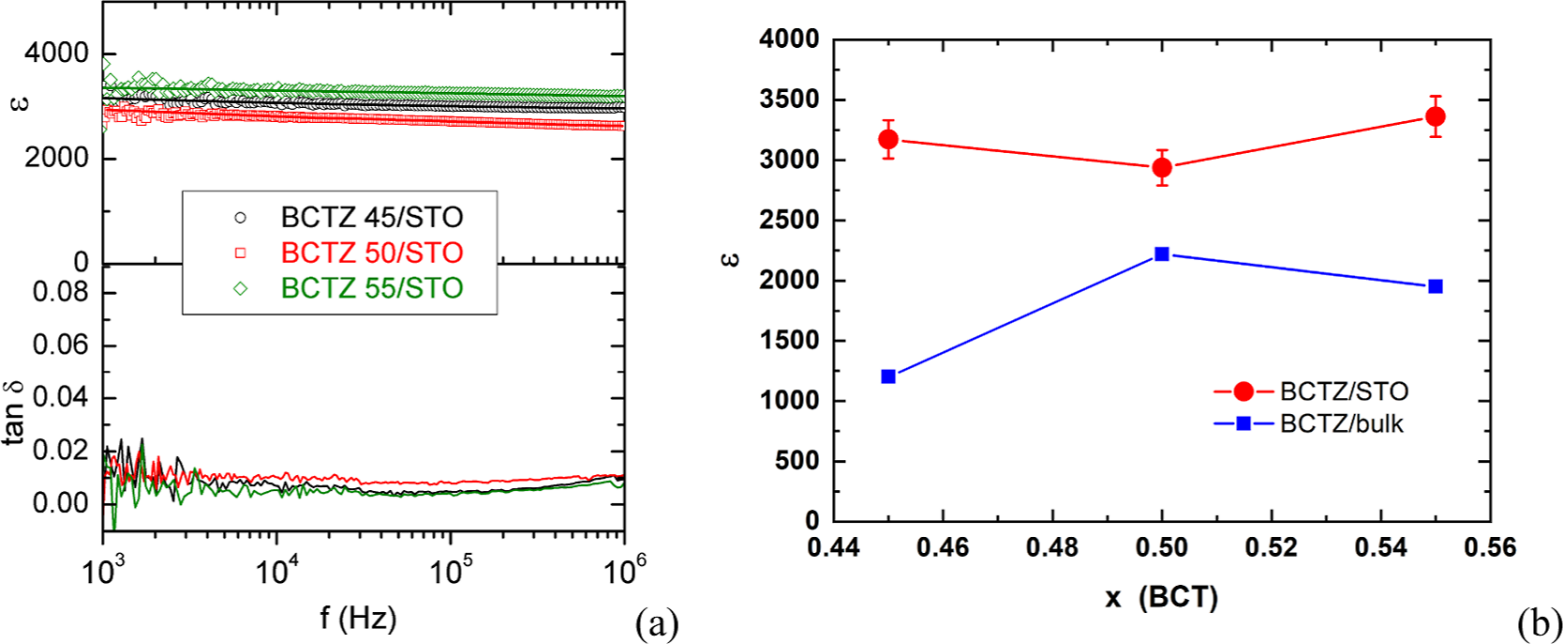
(a) BCTZ45/STO, BCTZ50/STO, and BCTZ55/STO dielectric permittivity and loss value dependence on frequency at room temperature. The errors are about 5%, but in order to avoid confusion, the error bars have not been inserted in the plot; (b) film dielectric permittivity (at 1 kHz) dependence on composition (the errors are about 5%). The lower plot represents the corresponding bulk values.

The dielectric permittivity of the film samples has been evaluated by using gold interdigital electrodes (IDEs) deposited on the top surface of the film. Dielectric measurements have been carried out on 2–9 different capacitors, depending on the type of material. The displayed results are for representative sets for each type of material. The measurements yield the capacitance and the dielectric loss tan δ between 1 kHz and 1 MHz, at ambient temperature. From capacitance values, the in-plane dielectric permittivity has been calculated according to the procedure described in Supporting Information, Note 4. The obtained values are plotted in Figure 4a. Although a dielectric permittivity dependence on frequency would be expected due to the relaxation of ferroelectric nanodomains, measurements at room temperature do not evidence it, since presumably they probe a region outside the dielectric anomaly with frequency dispersion.

The dependence on composition of film dielectric permittivity, measured at 1 kHz, is shown in Figure 4b. For comparison, bulk dielectric permittivities are plotted too. Very high values of relative dielectric permittivity are obtained for BCTZ 45 (around 3200) and BCTZ 55 (around 3400), while a slightly lower value (around 3000) has been measured on the BCTZ 50 film. All the films also show low dielectric loss (below 0.02) up to at least 1 MHz, due to their high quality and absence of defects.

When compared with the bulk permittivity values, which are much lower, a striking difference is noted in their dependence on composition, which is peaked at MPB *x* = 0.5 for the bulk samples, while it shows only a slight variation for film samples. For bulk samples, the maximum at MPB has been attributed to the coexistence of different phases and the presence of nanopolar regions.^27,28^ For film samples, we have explained the high values of dielectric permittivity for BCTZ 45 by the presence of nanopolar regions even at this composition below MPB, due to strain relaxation.^30^ It has been previously shown that the BCTZ compositions have the potential to transform their ground states under an applied mechanical constraint. For example, when a large compressive strain occurs in a thin film due to the epitaxial constraint from the underlying substrate, the ground state R transforms into a T-like (or O-like) phase, and upon partial strain relaxation in thicker films, this induced T phase evolves into a nanoscale mixture of R-like phase embedded into the T-like phase.^36^ Indeed, by GPA and HRTEM, the presence of nanodomains with various c/a tetragonality in BCTZ 45/STO films has been evidenced.^30^ The high dielectric permittivity of BCTZ 45 films was attributed to their high structural quality and to the presence of nanodomains, which favors the rotational instability of polarization.^37^

In Supporting Information, Note 2, we have shown HAADF–STEM images taken on BCTZ55, BCTZ50, and BCTZ45 films, which have been drift-corrected for GPA (Supporting Information, Figures S3–S5). For BCTZ55 and BCTZ45 films, the GPA strain maps evidenced a nanoscale strain variation, superposed on an inhomogeneous average strain, stronger near the interface film/substrate and more relaxed near the top surface. Instead, for the BCTZ50 film, the GPA strain maps evidence a much finer nanoscale variation and a minor variation of the average strain. Analysis of the strain profiles along the vertical of the film for in-plane (ε_*xx*_) and out-of-plane (ε_*zz*_) strains show that the misfit strain relaxes from the interface with the substrate toward the top surface (Supporting Information, Figures S6a–c). However, for the BCTZ50 film (Supporting Information, Figure S6b), after the initial partial relaxation, the strain changes are smaller and the structure remains slightly tetragonal (with small nanoscale fluctuations) with the same orientation [001] parallel to the normal. It seems that the rich nanoscale structure of all films is responsible for their high dielectric permittivity values, and it is the play between the chemical composition and peculiar strain relaxation that ultimately imparts the specific differences between permittivity values measured on different films.

### Determination of Pockels and Kerr Effects

The investigations of the electro-optic behavior of BCTZ/STO thin films were made using reflection-type SE measurements. Details about the measurement technique and evaluation of results are given in Methods and Supporting Information, Note 5. A transparent and conductive top electrode (Al-doped ZnO, AZO) was deposited on the top surface of BCTZ/STO (with 0.7 wt % Nb doping) films. Details about the obtaining of the heterostructure are given in Methods. The electrical resistivity of AZO measured by the four-point method has been around 7.8 × 10^–3^ Ω cm. The measurement of ellipsometric parameters of the heterostructures (Methods) has been used to extract the refractive indices and extinction coefficients for the BCTZ thin films, by employing WVASE32 software, which is designed to handle data modeling and fitting of complex multilayer problems.

The birefringence values in the absence of an electric field have been obtained by measuring the phase shift between p-polarized and s-polarized light components after the reflection on the sample, at different wavelength values, between 300 and 1200 nm. Then, the measurements in function of the electric field have been made by choosing a specific wavelength (λ = 500 nm) and an angle of incidence of 60°. The experimental phase shift values for different compositions have been further used to obtain the birefringence δ*n* values (Supporting Information, Note 5).

**Figure 5:**
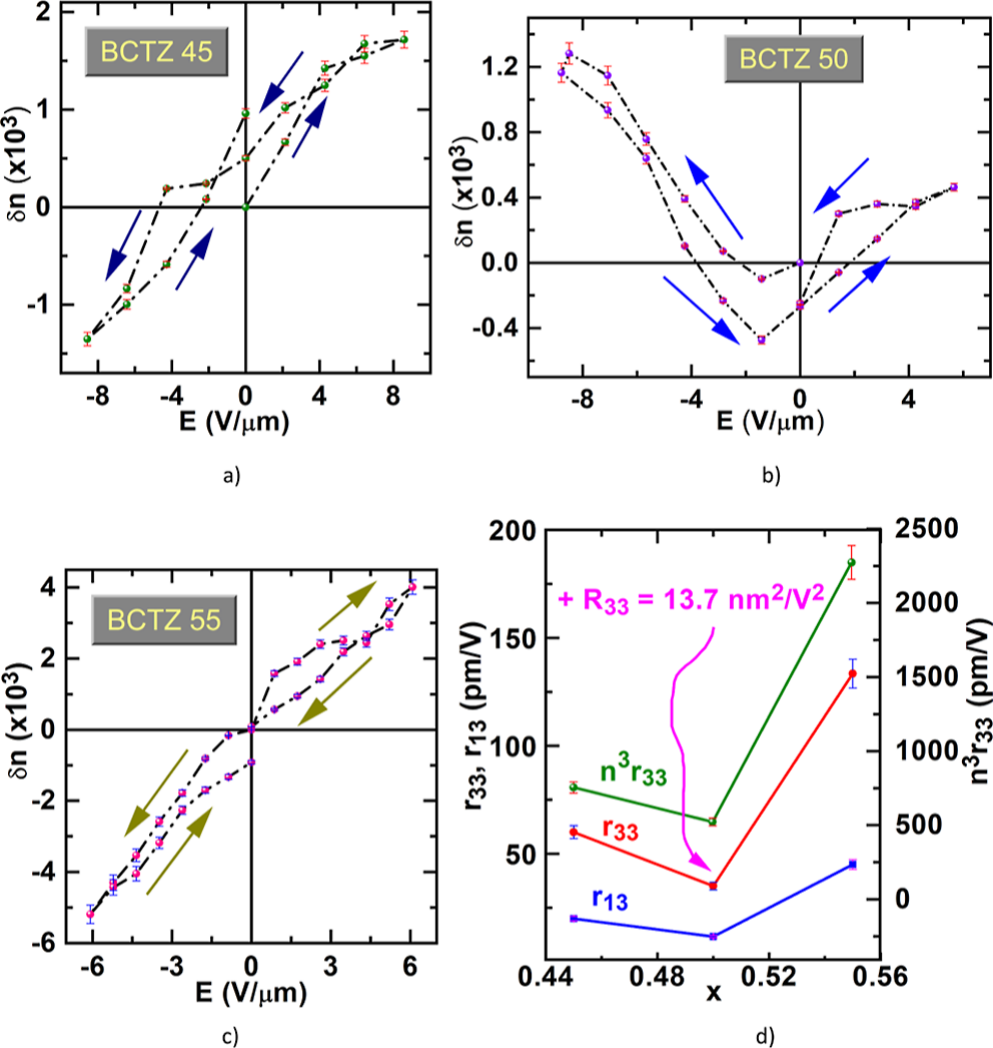
Birefringence shift dependence on the electric field for (a) BCTZ 45 films; (b) BCTZ 50, and (c) for BCTZ 55 films (the arrows indicate increasing or decreasing of the electric field). (d) Dependence of Pockels *r*_33_ and *r*_13_ coefficients and factor of merit *n*^3^*r*_33_ on composition. For *x* = 0.5 also a quadratic electro-optic coefficient is measured (indicated as *R*_33_ in the figure).

The obtained δ*n* values for different values of the applied electric field *E* are represented in Figure 5a–c. Their dependence on the electric field evidences a linear electro-optic behavior for BCTZ45 and BCTZ55 films, and a quadratic electro-optic behavior for the BCTZ50 film.

Pockels coefficients for BCTZ45 and BCTZ55 films have been extracted by fitting parts from the δ*n*(*E*) dependence by a linear function. The slope of the dependence *δn*(*E*) = *n*^3^*r*_eff_*E*/2, namely *n*^3^*r*_eff_/2, was used to calculate *r*_eff_ for the different films. Since in the ellipsometric experiment the probing beam was incident at θ = 60°, for a *c*-axis oriented thin film *r*_eff_ can be expressed as combination of *r*_33_ and *r*_13_, given by the relation^38^. Since it is difficult to assess *r*_33_ and *r*_13_ independently, we resort to an approximation valid for other perovskites like e.g., BaTiO_3_ (where *r*_33_ = 23 pm/V and *r*_13_ = 8 pm/V^39^), that is *r*_13_ ≅ 1/3 *r*_33_. Thus, in our case, for incidence at 60°, we have *r*_33_ ≅ 1.136 × *r*_eff_ (Supporting Information, Note 5). The obtained values are listed in Table 2. We must mention that in our electro-optical measurement configuration only the *r*_33_ and *r*_13_ coefficients are involved (ref (40).)

**Table 2:** Electro-Optic Linear and Quadratic Coefficients for Different Compositions^a^

sample	Refr. Ind. *n*	thick (nm)	*r*_eff_ (pm/V)	*r*_33_ (pm/V)	*r*_13_ (pm/V)	factor merit *n*^3^ × *r*_33_	*R*_eff_ (nm^2^/V^2^)
BCTZ 45	2.325	466.2	52.8	60	20	754.1	
BCTZ 50	2.46	706	30.86	35.1	11.7	522	13.7
BCTZ 55	2.56	575	119.3	135.5	45	2273.3	
BaTiO_3_^39^	2.365			23	8	334	
LiNbO_3_^39^	2.20			30.8	8.6	328	
BaTiO_3_^14^			30				
BaTiO_3_^14^			148*				
BaTiO_3_^15^			380*				
BaTiO_3_^18^			213*				
BaTiO_3_^17^			131*				
BaTiO_3_^17^			157*				
BaTiO_3_^43^			140*	20			
BaTiO_3_^43^			37*	16			
BaTiO_3_^19^			268*				

a For comparison, the corresponding coefficients for LiNbO_3_ and BaTiO_3_ have also been included.

Although the off-diagonal Pockels terms *r*_42_ (or *r*_51_) are the largest parameters in the Pockels matrix, the knowledge of the former ones is useful for applications where polarization rotation must be avoided, since they only induce changes in the diagonal terms of the permittivity matrix and therefore maintain polarization.

When comparing the effective electro-optic coefficients *r*_eff_ between different reported results, one must take into account that coupling the applied electric field to the *r*_42_ coefficient results into a highly increased *r*_eff_, due to the large value of the *r*_42_ term (in BTO this is nearly 6 times larger than *r*_33_).

Let us discuss now the behavior of BCTZ 50 thin films. Materials exhibiting quadratic electro-optic effect do not possess permanent polarization but exhibit substantial induced polarization when subjected to an electric field. This electrically induced polarization effect is actually an electrically induced phase change from the paraelectric, optically isotropic state to the FE, optically active, anisotropic state. The electrically induced birefringence of such a material is a quadratic form of the applied field (Kerr effect).^1^ For the Kerr effect, the refractive index change under an applied electric field is given by^1^
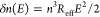
where *R*_eff_ is the effective Kerr coefficient.

However, as apparent from Figure 5b, for BCTZ 50 a linear effect is also present, due to the remnant polarization induced by the applied electric field (recall that the material possesses a nanoscale anisotropy). This is manifested through the non-zero birefringence at the zero field. Thus, fitting of the curve has been tried with a polynomial of second order. However, the best fitting could be achieved with a third- order polynomial in powers of the electric field. This indicates the existence of a superior order nonlinearity above the Kerr effect. Such superior effects have been recently predicted for BaTiO_3_,^41^ where a behavior of the electro-optic tensor of the type *r*_33_ + *R*_33_E + *S*_333_*E*^2^, with *r*_33_ = 39.6 pm/V, *R*_33_ = −6.4 × 10^–20^ m^2^/V^2^, and *S*_333_ = 5.1 × 10^–29^ m^3^/V^3^ has been numerically found by a first-principles technique. The field-induced behavior of the frequency of some specific phonon modes and of some force constants of the Ti–Ti, O–O, and Ti–O bonds are found to be responsible for the nonlinear behavior of BTO. As BCTZ is similar to BTO as structure and main elements, we presume that this behavior could be present to some extent also in the different BCTZ compositions. It is evident that the MPB films, which are highly nonlinear, exhibit an extra component coming probably from the field behavior of nanopolar regions, which are easily orientable by an electric field but prone to return at the initial configuration upon the field removal. Instead BCTZ 45 and BCTZ 55 films are basically ferroelectrics with R and T structures, and although the presence of nanoscale structures is evidenced, their behavior is essentially linear electro-optic.

The presence of both Pockels and Kerr effects in the BCTZ 50 films could be very appealing for photonic chips based on cascaded Pockels and Kerr nonlinear optical effects for efficient frequency conversion, of interest for quantum communication applications.^42^

The obtained linear and quadratic electro-optic coefficients and the factor of merit^39^*n*^3^*r*_33_ for films with different compositions are represented in Figure 5d and also listed in Table 2. A strong linear electro-optic behavior is obtained for the BCTZ 55 thin film. This composition is on the right side of MPB, with a higher ratio of Ti/Zr and Ca/Ba, thus more ferroelectric and with a stronger linear electro-optic behavior. However, an important role could also be played by the microstructure: films with less defects show higher electro-optical response.^43^

## Conclusions

We show in this work that BCTZ films with various compositions can be epitaxially grown on SrTiO_3_ up to high thicknesses. We show that, depending on the Ba/Ca and Ti/Zr ratios, BCTZ films display high Pockels (χ^(2)^) or Kerr (χ^(3)^) optical nonlinearities. The relevant finding is that the linear and nonlinear electro-optical properties of BCTZ films can be tailored by changing the Ba/Ca and Ti/Zr ratio in the composition. Their linear properties are superior to LiNbO_3_ which is the material used for optical applications. Moreover, LiNbO_3_ is difficult to be grown as thin films (no epitaxial deposition has been reported). The epitaxial BCTZ thin films with variable BCT(*x*) content (which ultimately translates into variable Ba/Ca and Ti/Zr ratios) show linear EO properties for rhombohedral (*x* = 0.45) and tetragonal (*x* = 0.55) compositions and nonlinear EO properties for MPB composition (*x* = 0.50), where a mix of phases coexist at the nanoscale, rending it similar to a relaxor ferroelectric with nanopolar regions. The quadratic EO effect is attributed to the coexistence of nanopolar regions with different symmetries. These findings open the way to the efficient integration of different electro-optical devices (modulators, tuning elements, bistable switches, etc.), including χ^(2)^- and χ^(3)^-based elements for room-temperature photonic quantum processing.

## Methods

### Fabrication of BCTZ Films

For PLD deposition of thin films, ceramic targets with different BCTZ compositions were fabricated by the solid-state reaction method. The sintered targets were polished down to 3 mm thickness for removing the outer layer and were characterized by XRD to check the structure. The films have been deposited on single crystal STO(100) substrates. An excimer ArF laser working at 193 nm wavelength and a 5 Hz repetition rate was employed. The laser fluence was set at 2 J.5/cm^2^. BCTZ film growth was carried out under atomic oxygen at a pressure of 10 Pa and substrate temperature of 700 °C. To enable the electro-optical measurements, a layer of AZO has been deposited on the top surface of the films.

### Structural Characterization

The structural properties, the targets, and their derived thin films were investigated by XRD using a PANalytical X’Pert MRD system, in a divergent beam Bragg–Brentano configuration (λ = 1.5418 Å) for the targets, and a parallel beam configuration provided by a hybrid monochromator 2× Ge(220) asymmetric (λ = 1.540598 Å) for the thin films. For the refinement of the patterns, the HighScore software package provided by PANalytical was used. The scans have been made on nine samples of different BCTZ compositions (four samples of BCTZ45, two samples of BCTZ50, and three samples of BCTZ55).

### Transmission Electron Microscopy

Cross section samples for TEM have been prepared by mechanical grinding and ion milling. HAADF–STEM was carried out using an FEI Titan Themis^3^300 microscope operated at 300 kV. The STEM images have been drift-corrected for GPA. Drift-corrected Super-X energy-dispersive X-ray mapping furnished quantified maps and line profiles of compositions. The scans have been made on three samples of different BCTZ compositions (BCTZ45, BCTZ50, and BCTZ55).

### Dielectric Spectroscopy Measurements

For dielectric measurements, Au IDEs have been deposited on top surfaces of the films by employing the lift-off technique. The IDEs’ characteristic dimensions have been the following: number of finger pairs *N* = 21, finger length *L* = 464 μm, finger width *w* = 10 μm, interspace between fingers 10 μm, and distance between finger centers *D* = 20 μm. Low-signal dielectric spectroscopy measurements have been carried out by using an HP4194A impedance bridge equipped with a special holder for contacting the IDE electrodes, in the frequency range from 100 Hz up to 1 MHz. During the experiment, the ac voltage amplitude was kept at 0.02 V. The measurements yield the capacitance and the dielectric loss tan δ. The measured capacitance values have been used to calculate the dielectric permittivity as shown in Supporting Information, Note 4. In order to verify the correctness of the measurement method and of the calculation model, IDE structures have also been deposited directly on the STO substrate, and its dielectric constant has been measured and compared with datasheet value. The measurements have been made on six samples of different BCTZ compositions (two samples of BCTZ45, two samples of BCTZ50, and two samples of BCTZ55).

### Electro-Optic Measurements

The electro-optic behavior of BCTZ thin films has been characterized by reflection-type SE measurements using a Woollam Variable Angle Spectroscopic Ellipsometer (VASE) equipped with a high pressure Xe discharge lamp, which generates light in the spectral range 1–5 eV from the near-IR to the UV. A transparent and conductive electrode of AZO has been deposited by radio frequency-assisted PLD on top of BCTZ/STO (with 0.7 wt % Nb doping) structures, at a temperature of 450 °C, 5 Pa oxygen pressure, and 150 W radio frequency power. The electrical resistivity of the AZO layer, measured by the four-point method, has been around 7.8 × 10^–3^ Ω cm. An electric field has been applied between the AZO and STO electrodes of the AZO/BCTZ/STO heterostructures during the electro-optical measurements. The E-O measurements have been made on six samples of different BCTZ compositions (two samples of BCTZ45, two samples of BCTZ50, and two samples of BCTZ55). Standard ellipsometry measurements have been first performed from 300 nm up to 1200 nm without an applied electric field, at a fixed angle of incidence. In order to obtain the refractive index and extinction coefficient, as well as the thickness of the layers, WVASE32 software, designed to handle data modeling and fitting of complex multilayer problems has been used. The electro-optic response has been measured at a wavelength of 500 nm and an incidence angle of 60°. The measured phase shift values between p-polarized and s-polarized waves have been used to calculate the birefringence shift. In order to minimize the experimental error, the measurements of phase shift (δΦ) with the applied electric field were done in the dynamic mode. In this dynamic mode, one can measure the phase shift (δΦ) during a specific period of time (∼10–15 min/step) for each step of applied voltage and the final values were obtained by a linear fit for the specific interval. In this way, the standard deviation of δΦ has been obtained. The birefringence δ*n*(*E*) dependence and the electro-optic coefficient evaluation are discussed in Supporting Information, Note 5.

## Supporting Information Available

Experimental details, lattice parameters from X-ray diffraction, TEM data, compositional analysis and elemental mapping, dielectric permittivity and loss evaluation from measurements of interdigital capacitance, and evaluation of electrooptic coefficients from SE (PDF)
