# Supplementary material for: Lead-Free Perovskite Thin Films with Tailored Pockels-Kerr Effects for Photonics
Source: ACS Appl Mater Interfaces. 2023 Jul 27;15(31):38039–48. doi: 10.1021/acsami.3c06499 (PMC10416211; doi:10.1021/acsami.3c06499)
Supplement: Supplementary file 1 — am3c06499_si_001.pdf [file am3c06499_si_001.pdf]

Supporting Information

Lead-free perovskite thin films with tailored Pockels-Kerr effects for photonics.

Valentin Ion[†]*, Valentin Teodorescu*[‡]*, Ruxandra Birjega*[†]*, Maria Dinescu*[†]*, Christoph Mitterbauer*[₸]*, Ioannis Alexandrou*[₸]*, Ioan Ghitiu*[†°]*, Floriana Craciun*[⊥]*, Nicu D. Scarisoreanu*[†*]

[†] National Institute for Laser, Plasma and Radiation Physics, 409 Atomistilor, 077125 Magurele, Romania

[‡]National Institute of Materials Physics, 105 bis Atomistilor, 077125 Magurele, Romania

[₸] Thermo Fisher Scientific, Materials & Structural Analysis, De Schakel 2, 5651 GE Eindhoven, the Netherlands

[°] Faculty of Physics, University of Bucharest, Magurele, 077125, Romania

[⊥] CNR-ISM, Istituto di Struttura della Materia, Area della Ricerca di Roma-Tor Vergata, Via del Fosso del Cavaliere 100, I-00133 Rome, Italy

E-mail: nicu.scarisoreanu@inflpr.ro

Contents

Supplementary Note 1 – Lattice parameters from X-ray diffraction 2

Supplementary Note 2 – Transmission electron microscopy data 5

Supplementary Note 3 – Compositional analysis and elemental mapping 8

Supplementary Note 4 – Dielectric permittivity and loss evaluation from measurements of interdigital capacitance..11

Supplementary Note 5 – Evaluation of electrooptic coefficients from spectroscopic ellipsometry 12

References 13

Supplementary Note 1 – Lattice parameters from X-ray diffraction

The XRD patterns of the three $(1-x)Ba(Zr_{0.2}Ti_{0.8})O_3 - x(Ba_{0.7}Ca_{0.3})TiO_3$ (x=0.45; 0.50; 0.55) (BCTZ 100x) targets are presented in **Figure S1 (a)**. All the patterns are indexed as a pseudo-cubic lattice (S.G. *Pm-3m*) for the sake of simplicity and comparative evaluation. As discussed in the main text, the BCTZ solid solution exhibits a "tilted" morphotropic phase boundary (MPB) separating the rhombohedral (R) and tetragonal (T) phases by an intermediate orthorhombic (O) phase near x=0.5 at room temperature [Refs. 1-3]. The evolution from a rhombohedral (for x=0.45) to a tetragonal (for x=0.55) symmetry is evident for the $(200)_{pc}$ peak, showing the splitting of the singlet rhombohedral (202) peak to the tetragonal doublet (002)/(200)(**Figure S1 (b)**).

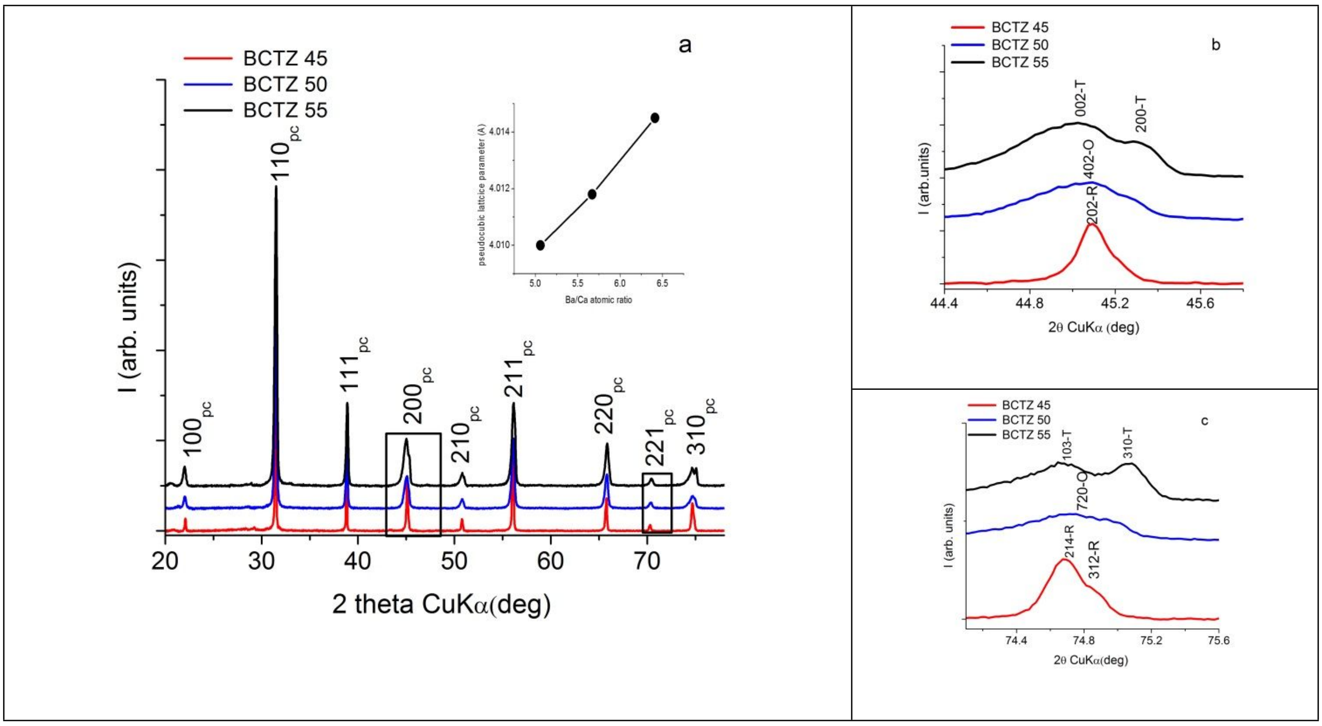

Figure S1. XRD patterns of the three BCTZ targets : (a) in the 2θ=20-80°; (b) 44.4-46° (c) 74.3-75.6° angular domains . Inset in (a): plot of the pseudo-cubic lattice parameter versus Ba/Ca atomic ratio.

Thus, the BCTZ45 target pattern was refined in a rhombohedral symmetry (S.G. *R*3*m*) and the BCTZ55 target pattern in a tetragonal symmetry (S.G. *P*4*mm*). The resulting crystallographic parameters were transformed into the equivalent pseudo-cubic parameters. The pseudo-cubic lattice constant (a_{pc}) represents the cube root of unit cell volume for ABO_3 [Ref. 4] . The obtained data are listed in **Table S1**. The BCTZ50 pattern displays very broad and ill-defined reflections as it can be observed in **Figure S1 (a-c)**. For example the $(200)_{pc}$ peak transforms from the singlet rhombohedral (202) peak for BCTZ45 to a very large

(402) orthorhombic peak for BCTZ50 or, in the case of $(310)_{pc}$ reflection, the double rhombohedral peak (214)/(312) of BCTZ45 merged in an extremely large orthorhombic peak for BCTZ50 (**Figure S1 (c)**).

The intermediate orthorhombic phase has actually multiple close diffractions peaks or might coexist with a rhombohedral phase or a tetragonal phase [Ref. 2]. Consequently, due to its limited accuracy, the BCTZ50 target profile was refined in a cubic symmetry (S.G. *Pm-3m*) in order to obtain the pseudocubic lattice constant. As expected, the pseudocubic lattice parameters exhibit a linear increase with increasing Ba/Ca ratios as a result of the larger ionic radius of Ba^{2+} (1.61 Å) with respect to Ca^{2+} (1.34 Å) (inset in **Figure S1 (a)**).

Table S1. Structural data of the BCZT targets

Target	Ba/Ca	Lattice parameters (Å)	Pseudocubic parameter (Å)
BCZT45	6.4	Rhombohedral (*R*3*m*)<br>*a*=5.679(1)<br>*c*=6.949(2)	4.0145
BCZT50	5.66	Orthorhombic (*Amm*2)<br>Refined as cubic *Pm*-3*m*<br>*a*=4.0118(5)	4.0118
BCZT55	5.0	Tetragonal (*P*4*mm*)<br>a=4.000(3)<br>c=4.030(3)	4.0100

For the BCTZ45, BCTZ50 and BCTZ55 films deposited on STO(001) the epitaxial growth was confirmed by the BCZT(101) and STO(101) Φ-scans (**Figure S2**), which evidences a 4-fold symmetry with 90^0 spacing for all the three films. The patterns were indexed as a pseudocubic lattice.

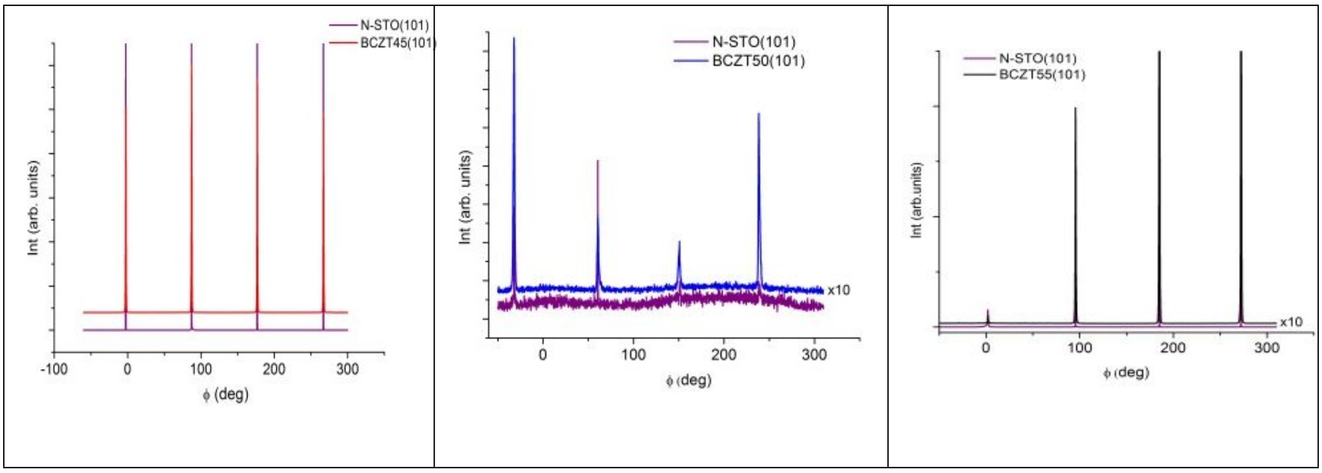

Figure S2. 360° φ-scans of BCZT (101) and STO (101) asymmetric reflections for the three BCZT films deposited on STO(001) substrate.

The out-of-plane (op) and in-plane (ip) lattice parameters (a_{op} and a_{ip}) for the deposited films were calculated from the symmetric and asymmetric XRD scans, respectively [Refs. 5-9].

The axial ratio (or tetragonal distortion) was calculated as the ratio a_{op}/a_{ip}. The out-of-plane and in-plane strains due to the misfit with respect to the substrate were calculated as follows:

$$\varepsilon_{zz} = \frac{a_{op} - a_{STO}}{a_{STO}} 100\% \quad (1)$$

$$\varepsilon_{xx} = \frac{a_{ip} - a_{STO}}{a_{STO}} 100\% \quad (2)$$

where a_{sto}=3.905 Å.

Supplementary Note 2 – Transmission electron microscopy data

High-angle annular dark-field (HAADF) scanning transmission electron microscopy (STEM) was carried out on BCTZ films. The STEM images have been drift-corrected for geometric phase analysis.

In **Figure S3**, we show a low-resolution HAADF-STEM image (**Figure S3 (a)**) and the corresponding geometric phase analysis (GPA) images for the strain along the $[010]_{pc}$ direction, and $[100]_{pc}$ direction, for the BCTZ55 film grown on STO substrate (Figure S3 (b,c)). The upper layer in the STEM image is the AZO conducting layer. The GPA strain maps corresponding to the rectangle area marked in the STEM image evidence a nanoscale strain variation, superposed on an inhomogeneous strain, stronger near the interface film/substrate and more relaxed near the top surface.

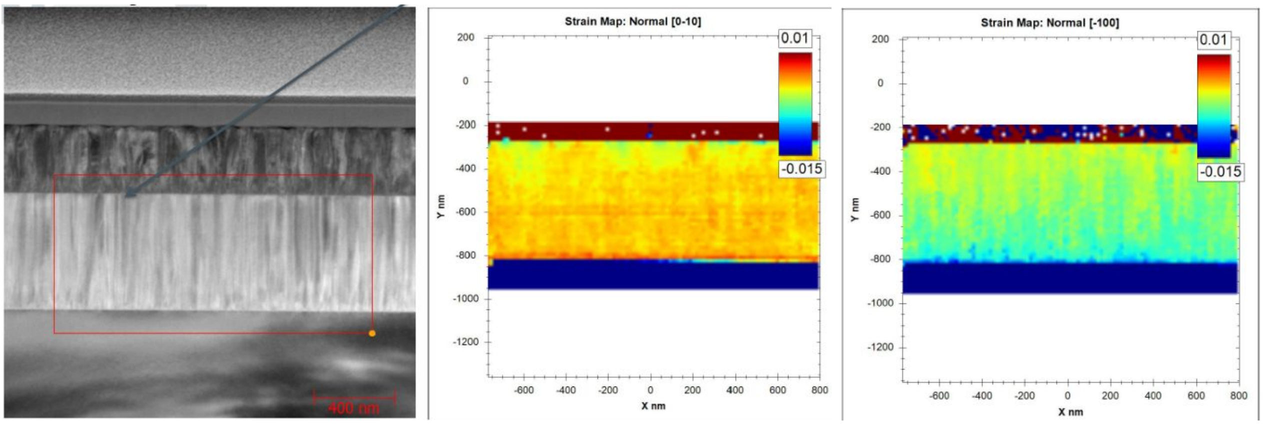

(a) (b) (c)

Figure S3. a) Cross-section low-resolution HAADF-STEM image for the BCTZ55 film grown on STO. The upper layer - AZO; b-c) GPA strain maps corresponding to the rectangle area marked in a) for the strain along the $[010]_{pc}$ and $[100]_{pc}$ directions, respectively.

In **Figure S4** a low-resolution HAADF-STEM image **(a)** taken on BCTZ50 film and the corresponding geometric phase analysis (GPA) images for the strain along the $[010]_{pc}$ direction, and $[100]_{pc}$ direction **(b-c)** are shown. The upper layer in the STEM image is the AZO conducting layer. The GPA strain maps corresponding to the rectangle area marked in the STEM image evidence a finer nanoscale strain variation.

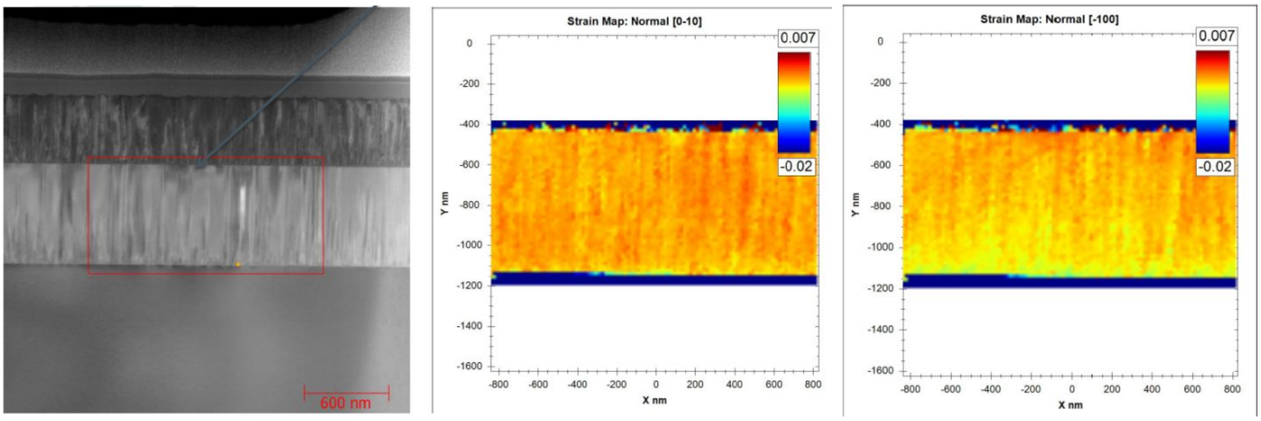

(a) (b) (c)

Figure S4. a) Cross-section low-resolution HAADF-STEM image for the BCTZ50 film grown on STO. The upper layer - AZO; b-c) GPA strain maps corresponding to the rectangle area marked in a) for the strain along the $[010]_{pc}$ and $[100]_{pc}$ directions, respectively.

In **Figure S5 (a)** a cross-section low-resolution HAADF-STEM image taken on BCTZ45 film grown on STO substrate (with an AZO layer on top). GPA images for the strain along the $[010]_{pc}$ direction, and $[100]_{pc}$ direction, corresponding to the area marked by a rectangle in (a), are displayed in (b) and (c), respectively. The GPA strain maps corresponding to the rectangle area marked in the STEM image evidence a fine nanoscale strain variation, superposed on an inhomogeneous strain, stronger near the interface film/substrate and more relaxed near the top surface.

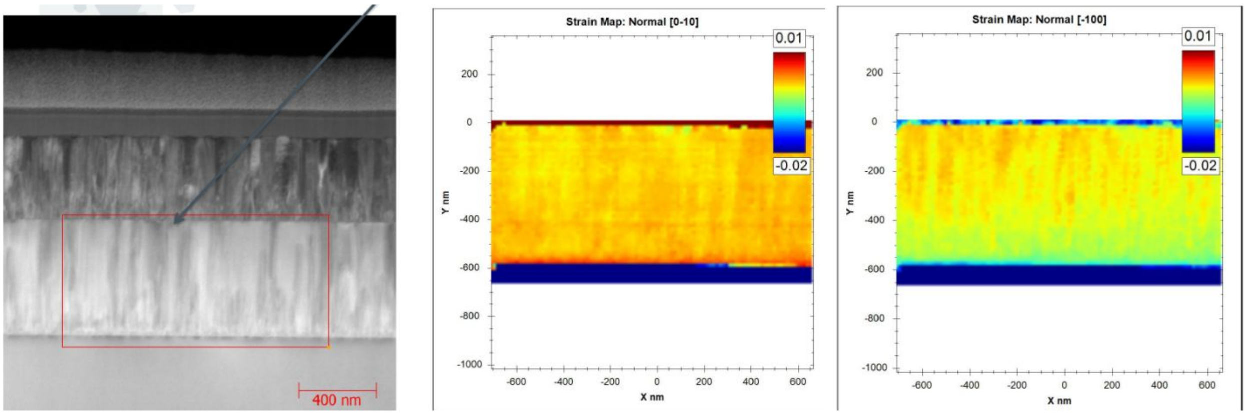

(a) (b) (c)

Figure S5. a) Cross-section low-resolution HAADF-STEM image for the BCTZ45 film grown on STO. The upper layer - AZO; b-c) GPA strain maps corresponding to the rectangle area marked in a) for the strain along the $[010]_{pc}$ and $[100]_{pc}$ directions, respectively.

Strain profiles along the vertical of the film for in-plane (ε_{xx}) and out-of-plane (ε_{zz}) strain show that the misfit strain relaxes from the interface with the substrate toward the top surface (**Figure S6 (a)-(c)**). The misfit strain is caused by the different lattice parameters of the STO(001) cubic substrate and the tetragonal (BCTZ55), orthorhombic (BCTZ50) or rhombohedral (BCTZ45) lattice parameters. The strain variation in the films generally reflects variations in the lattice parameters. The epitaxial films show different behavior for the different compositions. For example BCTZ50 film (**Figure S6 (b)**) remains slightly tetragonal (with small nanoscale fluctuations) with the same orientation [001] parallel to the normal. The BCTZ55 (**Figure S6 (a)**) and BCTZ45 (**Figure S6 (c)**) are tetragonal near the STO interface along [001] and gradually change, with small nanoscale fluctuations, to a tetragonal in-plane structure, towards the top interface. The arrows in the plots evidence different values of the out-of-plain lattice parameters, corresponding to the marked strains.

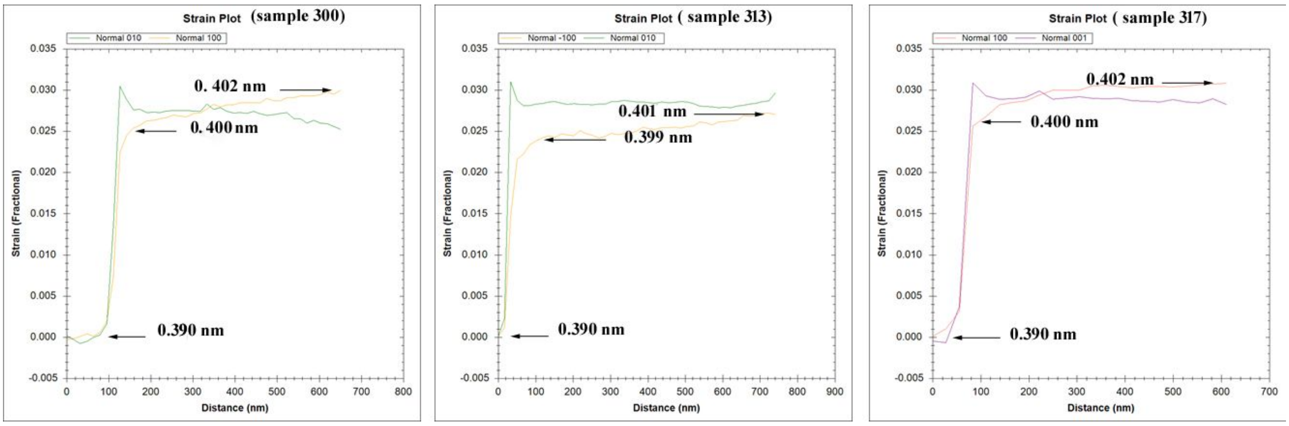

(a) (b) (c)

Figure S6. a) Strain profiles along the vertical of the film for in-plane (ε_{xx}) and out-of-plane (ε_{zz}) strain for the BCTZ55 film; (b) idem for the BCTZ50 film; (c) idem for the BCTZ45 film. The arrows evidence different values of the out-of-plain lattice parameters, corresponding to the marked strains.

Low-resolution HAADF-STEM images taken on BCTZ45 film (**Figure S7 (b)**) evidence a similar columnar aspect with BCTZ55 film (**Figure S7 (a)**) although, apparently, the density of columns seems higher in BCTZ55 than in BCTZ45. This can be related to a higher effective in-plane strain in BCTZ55, although the values listed in **Table 1 (manuscript file)** for the in-plane strain are rather similar. However, at a second look, one can notice that near the substrate interface the columnar density seems equally higher in both films, and that it is the behavior at some distance from the substrate which is different. This is correlated with in the in-plane coherence length values, extracted from XRD (**Table 1- manuscript file**) which are much higher in BCTZ45 film.

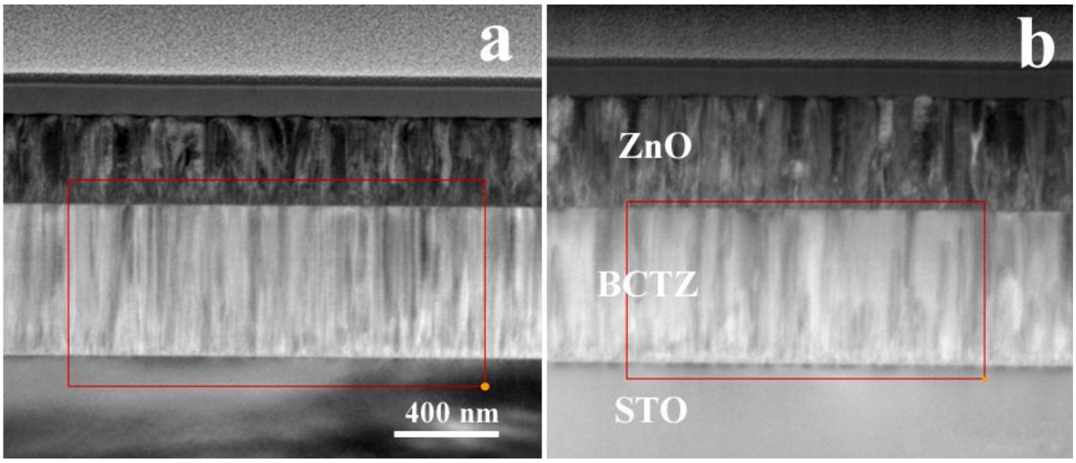

Figure S7. a) Cross-section low-resolution HAADF-STEM image for the BCTZ55 film grown on STO. The upper layer - AZO; b) idem for the BCTZ45 film.

Supplementary Note 3 – Compositional analysis and elemental mapping

Information about chemical composition on the nanoscale is important for the understanding of BCTZ film structure and physical properties. Eventual chemical inhomogeneities can bias phase structure and bring strong consequences on the physical properties. In order to verify film composition, extended maps of the elements have been obtained by Super-X energy-dispersive X-ray spectroscopy (EDX), which allows to acquire large area elemental maps with high spatial resolution and also light element sensitivity.

Figure S8 displays a comparison of the HAADF STEM image (left) with the quantified elemental mappings (right) extracted from the Super-X STEM EDX simultaneous measurements, for the elements present in the BCTZ55 film, the STO substrate and the AZO top layer. A uniform distribution of the main cations in the BCTZ55 film can be observed, with no penetration in the adjacent substrate and top layer. The oxygen element is distributed in all heterostructure, as a component of all oxide layers.

Super-X EDX quantified line profiles along the heterostructure normal for the elements present in the BCTZ55 film, the STO substrate and the AZO top layer are shown in **Figure S9**. This confirms the homogeneous average distribution of the elements along the thickness of the BCTZ55 film.

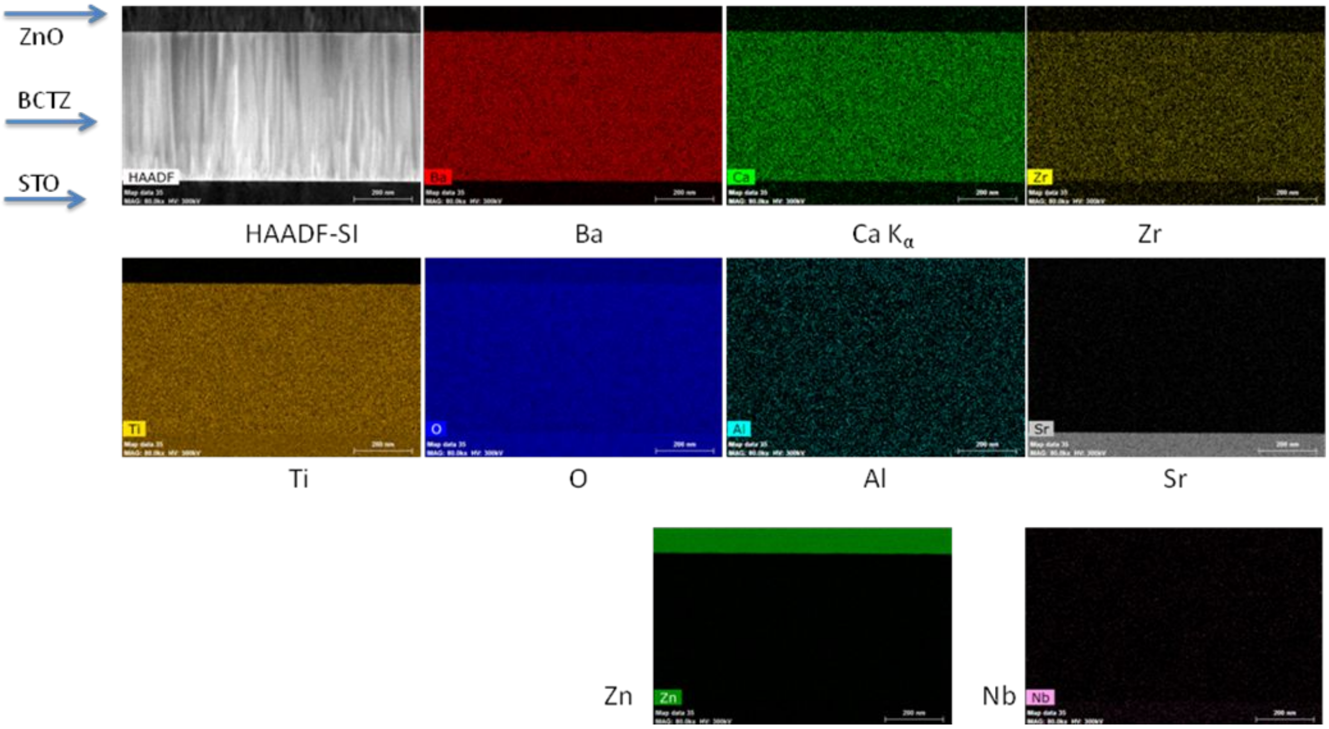

Figure S8. Cross-section low-resolution HAADF-STEM image and Super-X EDX mapping: quantified compositional maps for the elements present in the BCTZ55 film, the STO substrate and the AZO top layer.

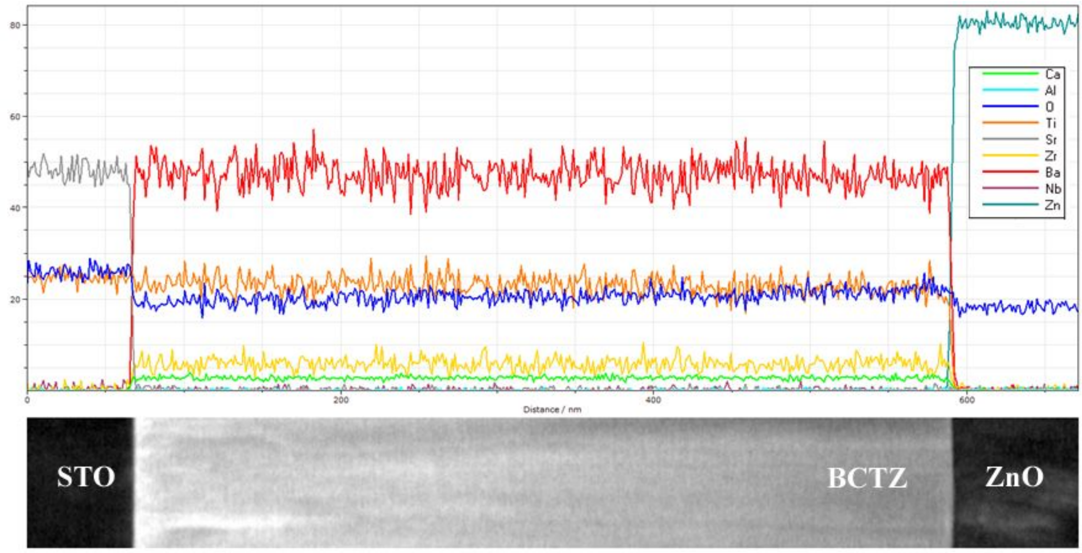

Figure S9. Super-X EDX quantified line profiles along the heterostructure normal for the elements present in the BCTZ55 film, the STO substrate and the AZO top layer.

The mapping of the elements have been also measured at atomic level. **Figure S10** shows an atomic resolution HAADF-STEM image and Super-X EDX elemental mapping of the BCTZ55 film near the top interface. The brighter spots in the left image represent the heavier Ba^{2+}/Ca^{2+} columns (the intensity is approximately proportional to the square of the atomic number). The central spots represent the light Ti^{4+}/Zr^{4+} columns (Ca and Zr are present in small amounts in the columns, therefore the contrast is conditioned by the majority Ba and Ti elements). A uniform distribution of the elements can be observed.

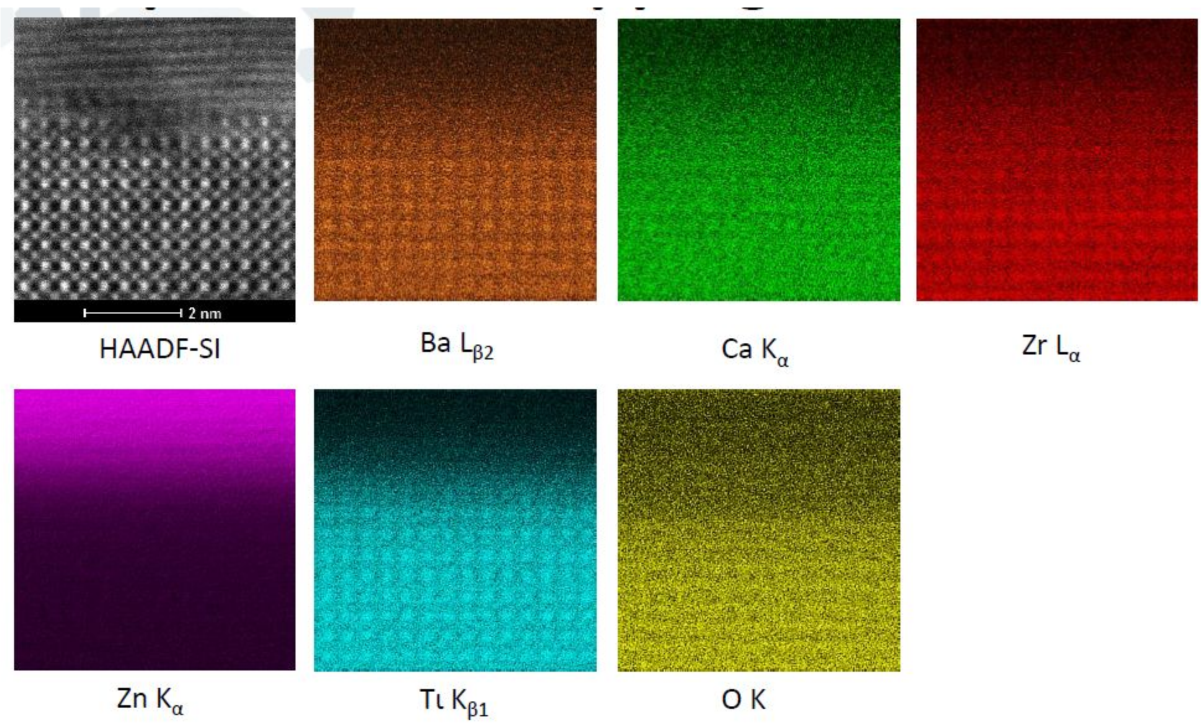

Figure S10. Atomic resolution HAADF-STEM image and Super-X EDX elemental mapping of the BCTZ55 film near the top interface. The brighter spots in the left image represent Ba/Ca columns, while the central spots represent Ti/Zr elements.

Figure S11 shows an atomic resolution HAADF-STEM image and Super-X EDX elemental mapping of the BCTZ50 film near the top interface. As in Figure S10, the brighter spots in the left image represent the heavier Ba^{2+}/Ca^{2+} columns (the intensity is approximately proportional to the square of the atomic number). The central spots represent the light Ti^{4+}/Zr^{4+} columns (Ca and Zr are present in small amounts in the columns, therefore the contrast is conditioned by the majority Ba and Ti elements). A uniform distribution of the elements can be observed.

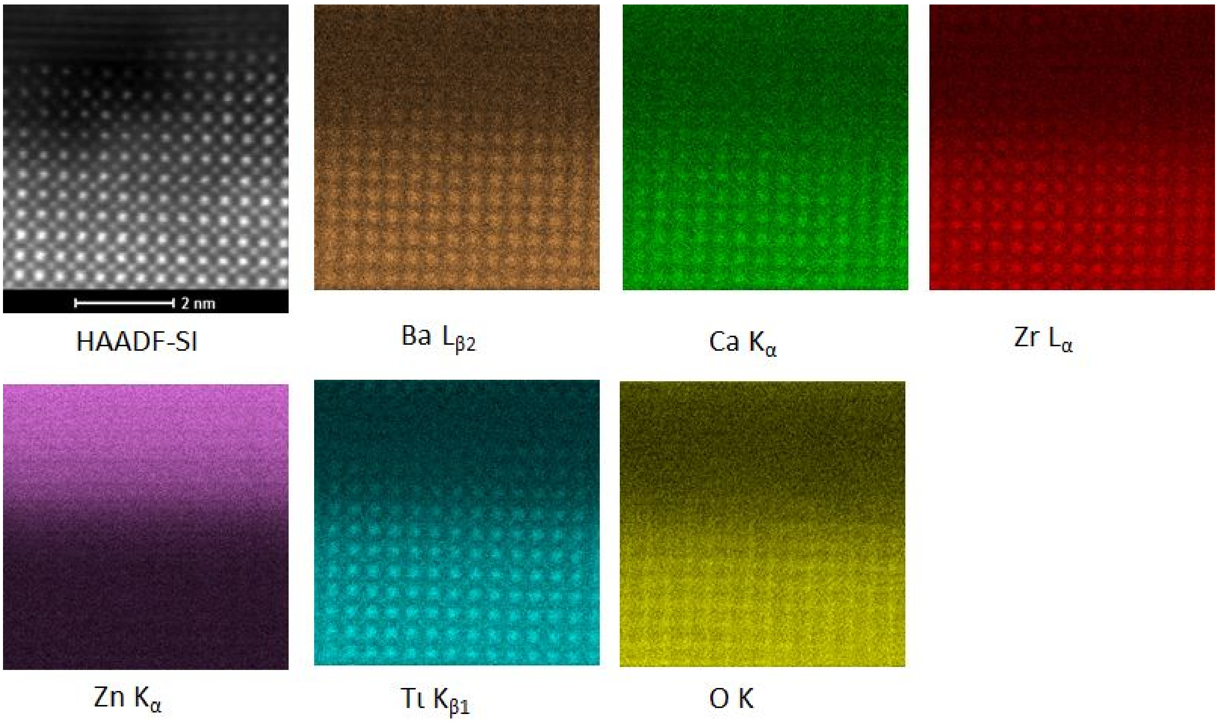

Figure S11. Atomic resolution HAADF-STEM image and Super-X EDX elemental mapping of the BCTZ50 film near the top interface. The brighter spots in the left image represent Ba/Ca columns, while the central spots represent Ti/Zr elements.

Supplementary Note 4 – Dielectric permittivity and loss evaluation from measurements of interdigital capacitance.

For the measurements of the capacitance C and dielectric loss tanδ gold interdigital electrodes (IDE) have been deposited on the BCTZ film surface by magnetron sputtering. The IDE structures consist of $N = 21$ finger pairs with length $L = 464$ μm and width 10 μm. The interspace between fingers was 10 μm, thus the distance D between finger centers was 20 μm.

The complex capacitance of the Au/BCZT/STO thin film capacitors with IDE configuration has been obtained by dielectric spectroscopy measurements carried out at ambient temperature between 1 kHz and 10 MHz yield.

The in-plane dielectric constants of the BCTZ45, BCTZ50 and BCTZ55 films have been calculated by using the analytical model proposed in Refs. [10-11], which gives for the dielectric constant of the thin film ε the following formula

$$\varepsilon = \varepsilon_S + \frac{C_n - C_K(1+\varepsilon_S)}{C_K(1-\exp(-4.6h/D))} \quad (3)$$

where ε_s is the dielectric constant of the substrate, h is the film thickness, C_n is the measured capacitance of the IDE structure normalized to the finger length (L) and to the number of fingers ($2N$-1), while C_K is a constant depending on IDE geometry. In the case of IDE patterns with equal finger width and spacing, C_K = 4.53 pF/m (Refs. [10-11]). The dielectric constant of the STO substrate is ~300, as reported in the datasheet. However we have also measured this value, by depositing IDE structures on the surface of the STO substrates and applying the same model to extract the dielectric permittivity from the measured capacitance.

Supplementary Note 5 - Evaluation of electrooptic coefficients from spectroscopic ellipsometry

The change or refraction index due to electric field δn (E) is related to the voltage-induced wavelength shift by the relation [Ref. 12]:

$$\delta n(E) = \frac{n}{\lambda}\delta\lambda(E) \quad (4)$$

From the variation of phase $\Phi = 2\pi d/\lambda$, where d is the path, one can find the relationship between the phase shift (expressed in radians) and the wavelength shift:

$$\delta\Phi(E) = 2\pi d\frac{\delta\lambda(E)}{\lambda^2} \quad (5)$$

Then the wavelength shift can be expressed as:

$$\delta\lambda(E) = \frac{\lambda^2}{2\pi d}\delta\Phi(E) \quad (6)$$

Thus the relationship between the refraction index shift and the phase shift is obtained as:

$$\delta n(E) = \frac{n\lambda}{2\pi d}\delta\Phi(E) \quad (7)$$

The spectroscopic ellipsometry measurements under applied electric field for revealing the electro-optic effects have been performed at wavelength 500 nm and an angle of incidence 60^0, as described in Methods. The measured phase shift values have been used to calculate the birefringence shift δn, according to the equation (7). Furthermore we obtained the electro-optic coefficients by fitting significant parts of the graphs of birefringence shift δn(E) with polynomials of first degree (for BCTZ 45 and BCTZ 55) and of third degree (for BCTZ 50). The coefficients of the polynomials have been used to calculate the electro-optic coefficients as further described.

For the Pockels effect the refractive index change under an applied electric field is given by [Lines-Glass]:

$$\delta n(E) = \frac{n^3 r_{eff} E}{2} \quad (8)$$

where r_{eff} is the effective electro-optic coefficient. Thus, the slope of the linear dependence $\delta n(E)$ is used to calculate r_{eff}.

Since in the ellipsometric experiment the probing beam was incident at $\theta = 60^0$, for a c-axis oriented thin film r_{eff} is given by a combination of r_{33} and r_{13}, given by the expression [Ref. 12]:

$$r_{eff} = (r_{13}^2 \cos^2\theta + r_{33}^2 \sin^2\theta)^{1/2} \quad (9)$$

Since it is difficult to assess independently r_{33} and r_{13}, we resort to an approximation valid for other perovskites like e. g. $BaTiO_3$ (where r_{33} = 23 pm/V and r_{13} = 8 pm/V ([Ref. 13]), that is $r_{13} \cong 1/3\ r_{33}$. In our case, for incidence at 60^0, we have

$$r_{eff} \cong (\frac{1}{9} r_{33}^2 \cos^2\theta + r_{33}^2 \sin^2\theta)^{1/2} = r_{33}(\frac{1}{9}\cos^2(\pi/3) + \sin^2(\pi/3))^{1/2} \cong r_{33} \times 0.88 \quad (10)$$

and then

$$r_{33} \cong 1.136 \times r_{eff} \quad (11)$$

For the Kerr effect, the refractive index change under an applied electric field is given by [Lines and Glass]:

$$\delta n(E) = \frac{n^3 R_{eff} E^2}{2} \quad (12)$$

where R_{eff} is the effective Kerr coefficient.

For the BCTZ50 thin film the best fitting has been achieved with a polynomial of third degree. From the fitting parameters the Pockels and Kerr effective coefficients have been extracted.

References

1. Keeble D. S., Benabdallah F., Thomas P. A., Maglione M. & Kreisel J. Revised structural phase diagram of $(Ba_{0.7}Ca_{0.3}TiO_3)$-$(BaZr_{0.2}Ti_{0.8}O_3)$. *Appl. Phys. Lett.* **102**, 092903 (2013).

2.Y. Tian, L. Wei, X. Chao, Z. Liu, Z. Yang, J. Am. Ceram. Soc., 96(2), (2013), 496-502.

3. M. Acosta, N. Novak, V. Rojas, S. Patel, R. Vaish, J. Koruza, G. A. Rossetti, Jr., J. Rödel, Appl. Phys. Rev.4, (2017), 041305

4. N. Dasgupta, R. Krishnamoorthy , K. Thomas Jacob, Mater.Sci .Eng.,B, 90, 278-286, (2002)

5. N. D. Scarisoreanu et al. J.Appl.Phys. 116,(2014), 074106-7

6. Scarisoreanu N. D. et al. High Permittivity $(1 - x)Ba(Zr_{0.2}Ti_{0.8})O_3$-$x(Ba_{0.7}Ca_{0.3})TiO_3$ (x = 0.45) Epitaxial Thin Films with Nanoscale Phase Fluctuations. *ACS Appl. Mater. Interfaces* **7**, 23984−23992 (2015).

7. T. Metzger, R. Höpler, E. Born, O. Ambacher,W. Stutzmann,R. Stömmer,W. Schuster, H. Göbel, S. Christiansen, W.Albrecht, M.P. Strunk, Philos. Mag. A, 77, (1998), 1013-1025

8. R. Chierchia, T. Böttcher, H. Heinke, S. Einfled, S. Figge, D. Hommel, J. Appl. Phys, 93, (2003), 8918-8925

9. Scarisoreanu N. D. et al. Joining Chemical Pressure and Epitaxial Strain to Yield Y-doped BiFeO3 Thin Films with High Dielectric Response. *Sci. Rep.* **6**, 25535 (2016).

10. Farnell, G. W.; Cermak, I. A.; Silverster, P.; Wong, S. K. Capacitance and Field Distributions for Interdigital Surface-Wave Transducers. IEEE Trans. Sonics Ultrason. 1970, SU-17, 188−195.

11. Al-Shareef, H. N.; Dimos, D.; Raymond, M. V.; Schwartz, R. W.; Mueller, C. H. Tunability and Calculation of the Dielectric Constant of Capacitor Structures With Interdigital Electrodes. J. Electroceram. 1997, 1, 145−153.

12. Akazawa H. and Shimada M. Electro-optic properties of *c*-axis oriented $LiNbO_3$ films grown on Si(100) substrate. Materials Science and Engineering B 120 (2005) 50–54.

13. Yariv A. *Quantum Electronics* (John Wiley & Sons, New York, 1989).
